# Insulin-Like Growth Factor 2 mRNA-Binding Protein 3 and Its Related Molecules as Potential Biomarkers in Small-Cell Lung Cancer

**DOI:** 10.1155/2022/5774339

**Published:** 2022-07-07

**Authors:** Lin Zhu, Xing Gao, Yun Du, Min Tang, Xiaofei Guo, Zhigang Chen, Yulong Liu, Yimin Lu

**Affiliations:** ^1^Department of Respiratory, Kunshan First People's Hospital, Suzhou 215004, China; ^2^Department of Oncology, The Second Affiliated Hospital of Soochow University, Suzhou 215004, China; ^3^Department of Ultrasound, The Second Affiliated Hospital of Soochow University, Suzhou 215004, China

## Abstract

**Background:**

Insulin-like growth factor 2 mRNA-binding protein 3 (IGF2BP3) plays a key role in tumorigenesis and tumor progression. Lung cancer is the leading cause of cancer-related death in men and women all over the world. However, the relationship between IGF2BP3 and small-cell lung cancer (SCLC) has not been reported yet.

**Methods:**

SCLC and normal samples (GSE19945 and GSE149507) were obtained in the Gene Expression Omnibus (GEO) dataset. Differential genes were screened by R software, and functional analysis and signal pathway enrichment analysis were carried out. In addition, we used the survival analysis database to analyze the relationship between prognosis and gene expression. Besides, immunohistochemistry (IHC) and quantitative real-time PCR (qPCR) were used for further research.

**Results:**

Five differentially expressed miRNAs and 9 differentially expressed mRNAs were selected by using R software. Survival analysis database results show that C7, CLIC5, PRDX1, IGF2BP3, and LDB2 were related the overall survival of patients with SCLC. Furthermore, multivariate analysis included that IGF2BP3 was independent risk factors for SCLC patients. Besides, gene function and signal pathway enrichment analysis showed that differentially expressed miRNAs were involved in the process of tumorigenesis and development. Furthermore, IHC and qPCR outcomes showed that the expression level of hsa-miR-182, hsa-miR-183, and IGF2BP3 was differentially expressed in normal lung tissues (NLTs) and SCLC tissues (SCLCTs).

**Conclusions:**

Our results concluded that hsa-miR-182, hsa-miR-183, and IGF2BP3 may take part in the development of SCLC.

## 1. Background

Small-cell lung cancer (SCLC) accounts for about 15% of all lung cancer cases, which has very

high mortality on account of its high relapse rate after standard-of-care treatment, coupled with a deficiency of active second-line treatment [1]. It is a rapidly developing, neuroendocrine (NE) fatal lung cancer with a 5-year survival rate of 7%. Although targeted therapy and immunotherapy have significantly improved the prognosis of non-small-cell lung cancer (NSCLC), SCLC still cannot carry out precision medicine, and chemotherapy is still the basic treatment [2, 3]. Genomics research shows that there is no regulated gene mutation, and many clinical trials have failed to bring lasting clinical benefits [4]. Through the detection of related genes, predicting the efficacy of chemotherapy drugs and selecting appropriate drugs for individualized chemotherapy has become a reasonable choice to improve the efficacy and reduce ineffective treatment.

MicroRNAs (miRNAs) are small noncoding RNAs with a length of 20-22 BP, which are transcribed by the genome. miRNAs guide the silencing complex (RISC) to degrade mRNA or inhibit the translation of mRNA by pairing with the mRNA base of the target gene, to regulate protein expression at the transcriptional or posttranscriptional level [5]. Many special miRNAs are differentially expressed in tumor and normal tissues, such as miR-206, miR-141-3p, miR-30a, and miR-194-5p [6]. Krol et al. found that miR-182-5p inhibited ubiquitin-conjugating enzyme E2T protein expression by targeting ubiquitin-conjugating enzyme E2T mRNA and then inhibited the proliferation, migration, and invasion of tumor cell [7]. More interestingly, the differential expression of some miRNAs is related to different risk factors, such as miR-96 is related to HBV infection and miR-126 is related to alcohol consumption [8]. The difference of some miRNA expression profiles in liver cancer may be the result of malignant transformation of tumor cells, such as invasion, metastasis, and recurrence. For example, the abnormal expression of miR-151 leads to the downregulation of the downstream target gene Rho GDP dissociation inhibitor A (Rho GDP dissociation inhibitor A), which promotes the metastatic potential of liver cancer [9]. Recent studies have also found that nearly 80% of the discovered miRNAs related to tumor metastasis are related to the recurrence and survival rate of tumor patients [10]. Therefore, these miRNAs are of great significance in the occurrence and prognosis of tumor. These researches demonstrated that microRNA is closely related to the occurrence and development of tumors and is abnormally expressed in a variety of tumors. Controlling the expression of cancer-related miRNAs is expected to become a new generation of the drug model for the treatment of advanced cancer.

In this study, through the mining and analysis of database samples, we screened the differentially expressed genes in SCLC tissues to find potential biological targets for tumor therapy.

## 2. Methods

### 2.1. Database Screening

Bioinformatics is a subject that studies the collection, processing, storage, dissemination, analysis, and interpretation of biological information. It is also a new subject formed by the combination of life science and computer science with the rapid development of life science and computer science. Through the comprehensive utilization of biology, computer science, and information technology, it reveals the biological mysteries endowed by a large number of complex biological data. GSE19945 and GSE149507 were downloaded from the GEO database.

### 2.2. Difference Analysis

R language is a branch of s language, which is widely used in the field of statistics and was born around 1980. R is a complete software system for data processing, calculation, and drawing. Its functions include data storage and processing system, array operation tool, complete and coherent statistical analysis tools, excellent statistical mapping function, and simple and powerful programming language: it can manipulate the input and output of data, branch and cycle, and user-defined functions. We utilized limma R package to screen differentially expressed genes. |log2FC| > 1 and FDR < 0.05 were set as standards.

### 2.3. Gene Function and Signal Pathway Analysis

A biological process is usually participated in by a group of genes, rather than completed by a single gene alone. The basic premise of enrichment analysis is that if a biological process is abnormal in known studies, the genes that function together are likely to be selected as a set of genes related to this process. At present, the most common method based on GG annotation is to obtain a large number of functional overlapping results, which is not conducive to the further analysis of GG function. clusterProfiler package and Cytoscape were utilized for transcription factor, Gene Ontology, and Kyoto Encyclopedia of Genes and Genomes enrichment analysis.

### 2.4. MicroRNA and Target mRNA

MicroRNAs (miRNAs) are small endogenous noncoding RNA molecules composed of about 21-25 nucleotides. mRNA is short for messenger RNA, or messenger RNA. mRNA is transcribed from DNA and carries the corresponding genetic information to provide the required information for the next translation into protein. These small miRNAs usually target one or more mRNAs and regulate gene expression by inhibiting or breaking target mRNAs at the translation level. Using prediction software, we screened and analyzed the potential target genes of miRNA. Besides, GSE149507 was used for further study.

### 2.5. The Relationship between Identified Genes and Overall Survival of Patients with SCLC

KM plotter (http://kmplot.com/analysis/) is an online website for survival analysis. At present, the website can target 54675 genes and 18674 cancer samples, including breast cancer and lung cancer. The data types include chip data, high-throughput sequencing data, mRNA, and miRNA. The website is usually used to analyze the relevant research data (such as the data of geographic database and TCGA database), select biomarkers, and then use the website to analyze and verify the survival of the target gene, so as to prove and enrich the experimental results. Survival analysis was conducted by uploading the differentially expressed genes we selected into the website.

### 2.6. The Relationship between DEG Expression and Clinical Characteristics in TCGA

TCGA project was launched in 2006. After more than 10 years, the project has had a far reaching impact on global cancer research. The pan-Cancer Atlas Project is part of the TCGA project. The project analyzes 33 different cancer types from 11000 cases, so as to tell us the occurrence, where and why of human tumors. By analyzing the relationship between clinical data and gene expression in the database, we can study the impact of differential genes on the survival and prognosis of tumor patients.

### 2.7. Immunohistochemical Staining

The human protein atlas (HPA) (https://www.proteinatlas.org/) provides information on the tissue and cellular distribution of 26000 human proteins. The Knut and Alice Wallenberg foundation in Sweden established this database and used specific antibody and immunohistochemical techniques to study the distribution and expression of each protein in 48 normal human tissues, 20 tumor tissues, 47 cell lines, and 12 blood cells. The results showed at least 576 immunohistochemical maps, which were read and indexed by professionals. The tissues examined were from 144 different individuals and 216 tumor tissues, which ensured the full expression of staining results. This is a large-scale protein research project, which is mainly committed to mapping protein sites encoding genes expressed in human tissues and cells. We used this database to study the expression of the screened genes in SCLC.

### 2.8. Real-Time Quantitative Polymerase Chain Reaction

When SYBR Green I dye is added to the sample, it can immediately bind to the double stranded DNA in the sample. During PCR, the Applied Biosystems AmpliTaq Gold DNA polymerase can amplify the target sequence to produce PCR products, namely “amplicons.” The SYBR Green I dye then binds to each newly generated double-stranded DNA molecule. With the progress of PCR, more and more amplicons were generated. Since SYBR Green I dye can bind to all double stranded DNA, the fluorescence intensity will also increase with the increase of the PCR product.

## 3. Results

### 3.1. Identification of DEGs

**Figure 1:**
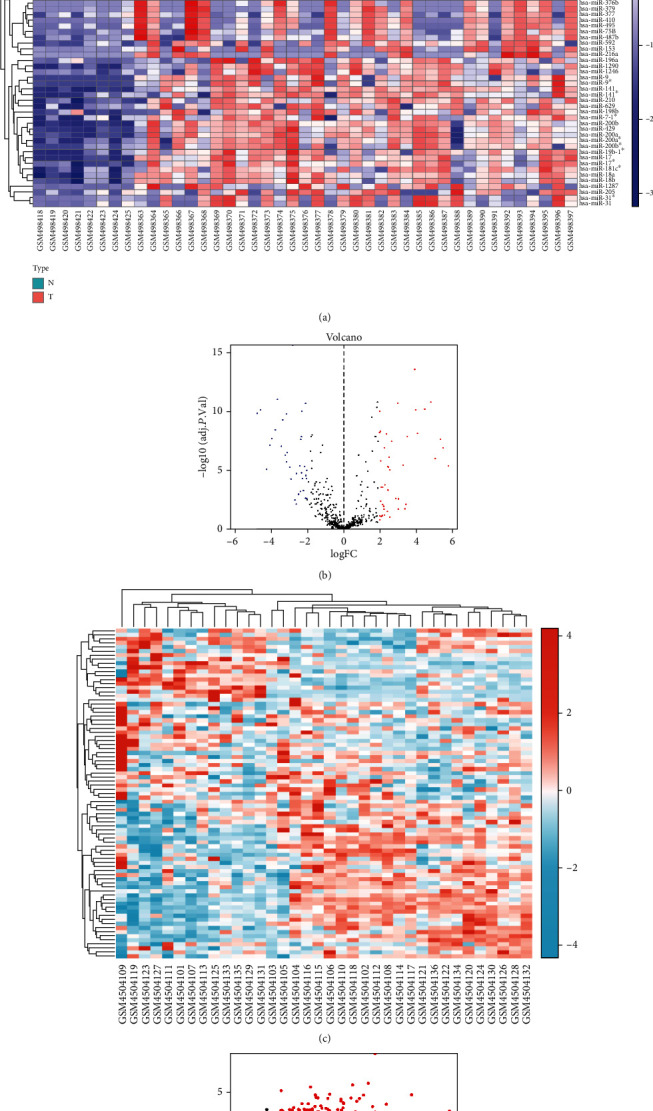
Heat map and volcano map of DEGs of GSE19945 and GSE149507. (a) Heat map of DEGs in GSE19945. (b) Volcano map of DEGs in GSE19945. (c) Heat map of DEGs in GSE149507. (d) Volcano map of DEGs in GSE149507. Red dots represent upregulated genes, and blue or green dots represent downregulated genes.

R software was utilized to research the differentially expressed genes from the GSE19945 and GSE149507. 82 DEMs (50 downregulated and 32 upregulated) including hsa-microRNA-9, hsamicroRNA-1290, hsa-microRNA-7, hsa-microRNA-183, hsa-microRNA-130b, hsa-microRNA-301b, hsa-microRNA-182, hsa-microRNA-144, hsa-microRNA-30a, and hsa-microRNA-1 and 102 DEGs (48 downregulated and 54 upregulated) were identified (Figure 1).

### 3.2. Gene Ontology Enrichment Analysis

**Figure 2:**
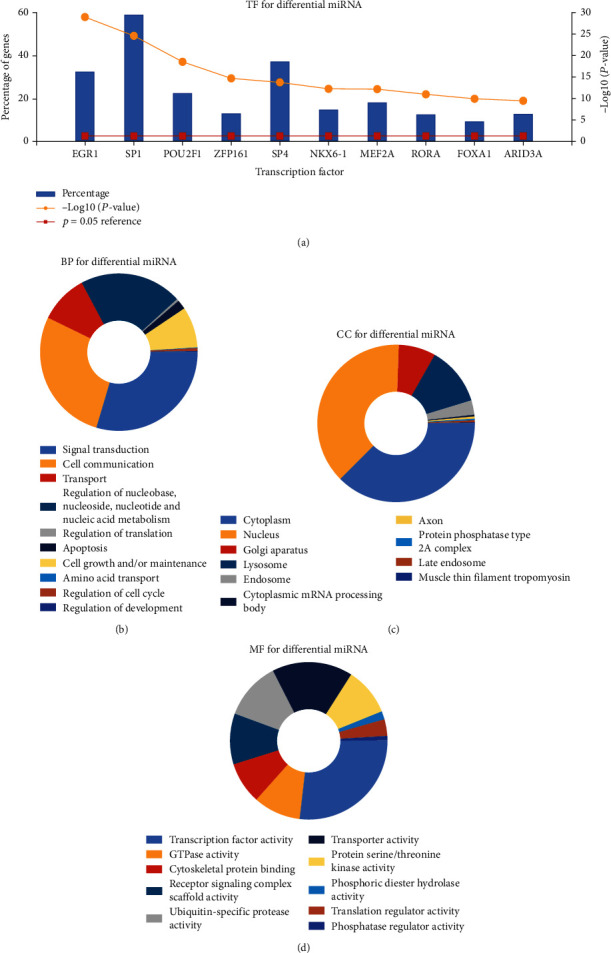
Gene ontology enrichment. (a) Transcription factor enrichment analysis of DEMs by FunRich software. (b) Biological process, (c) cellular component, and (d) molecular function enrichment analysis of the DEMs.

**Figure 3:**
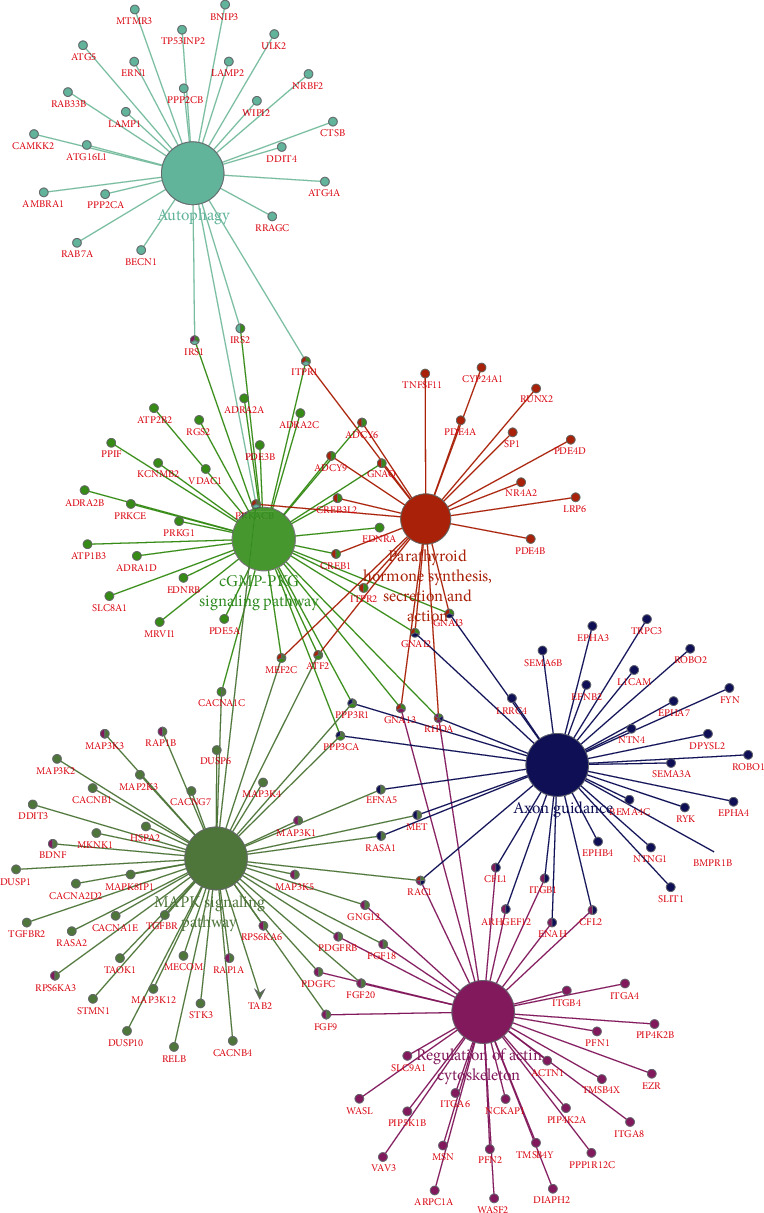
Kyoto Encyclopedia of Genes and Genomes pathway enrichment analysis of potential target mRNAs.

Gene function enrichment analysis refers to statistical analysis with the help of various databases and analysis tools to mine gene function categories that are significantly related to the biological problems we want to study in the database. Its statistical principle is to test the significance of a functional class in a group of genes (coexpression or differential expression) by hypergeometric distribution. Through the significance analysis, enrichment analysis, and false-positive analysis of discrete distribution, the functional classes of genes with significant correlation, low false positive rate and targeting can be obtained. Transcription factor enrichment analysis (Figure 2(a)) and GO analysis indicated that differentially expressed miRNAs had the most uniquely enriched terms for the cytoskeleton organization and biogenesis, cell cycle, cell growth and/or maintenance, lysosome, cytoplasmic mRNA processing body, axon, transporter activity, translation regulator activity, and protein binding (Figures 2(b)–2(d)). Furthermore, differentially expressed miRNAs were mainly enriched in 8 pathways: MAPK signaling pathway, cGMP-PKG signaling pathway, autophagy, axon guidance, regulation of actin cytoskeleton, parathyroid hormone synthesis, secretion and action, ErbB signaling pathway, and neurotrophin signaling pathway (Figure 3).

### 3.3. miRNA and Target mRNA

**Figure 4:**
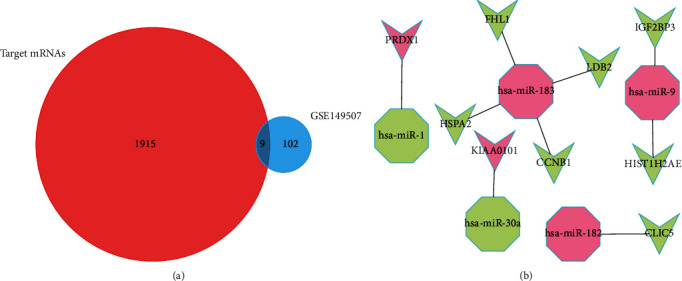
miRNA-mRNA network. (a) Venn Diagram of GSE19945 and GSE149507. (b) Identified target mRNAs and miRNA-mRNA regulatory network.

By using Prediction software, 1924 mRNAs were selected and 10 of them differentially expressed in GSE149507 (LDB2, KIAA0101, IGF2BP3, HSPA2, HIST1H2AE, FHL1, CLIC5, CCNB1, and PRDX1). According to the interaction relationship, 9 miRNA-mRNA pairs (microRNA-183, microRNA-9, microRNA-1, microRNA-30a, and microRNA-182) were identified for further study (Figure 4).

### 3.4. The Relationship between Gene Expression and SCLC Overall Survival

**Figure 5:**
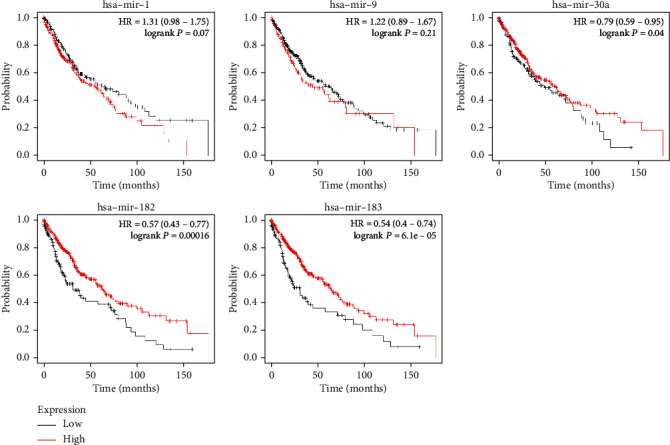
The relationship between the expression level of identified miRNAs and SCLC.

**Figure 6:**
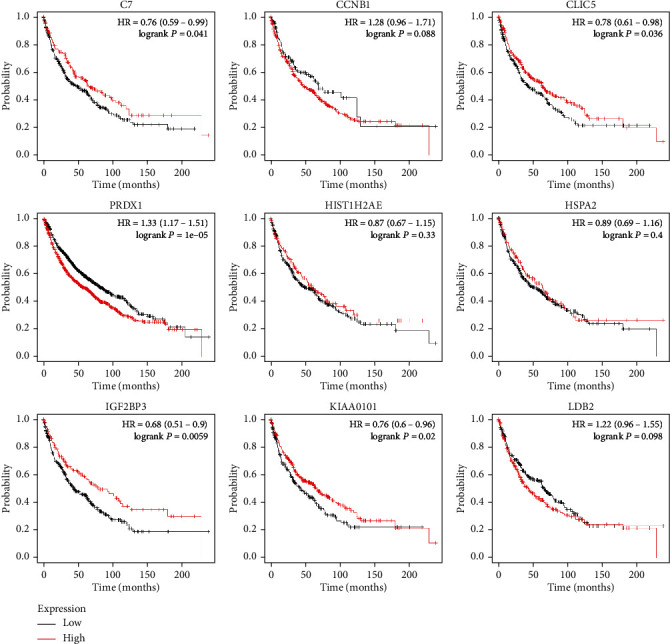
The relationship between the expression level of identified mRNAs and SCLC.

KM Plot was utilized to research SCLC overall survival. Results indicated that microRNA-182, microRNA-30a, and microRNA-183 (Figure 5) were related to the overall survival of patients with SCLC. Furthermore, the results of target genes also indicated that the expression level of C7, CLIC5, PRDX1, IGF2BP3, and KIAA0101 were significantly related to the overall survival of patients with SCLC (Figure 6).

### 3.5. Clinical Characteristics and mRNA Expression of SCLC

TCGA uses genome analysis technology based on large-scale sequencing to understand the molecular mechanism of cancer through extensive cooperation, improve people's scientific understanding of the molecular basis of cancer pathogenesis, and improve our ability to diagnose, treat, and prevent cancer. From the database, we can download gene mutation data, gene expression data, and patient clinical data. Through data processing, we found that the expression of IGF2BP3 was related to the stage of ccRCC patients, and the expression of IGF2BP3 in locally advanced patients was higher than that in early patients (Table 1). Besides, relationships between clinical characteristics and overall survival in SCLC were researched. Our results indicated that IGF2BP3 mRNA expression level (*p* = 0.006), lymph node stage (*p* < 0.001), metastasis stage (*p* = 0.008), and tumor stage (*p* < 0.001) were significantly associated with the OS of patients with SCLC (Table 2).

### 3.6. Validation of the Expression with qRT-PCR and IHC

**Figure 7:**
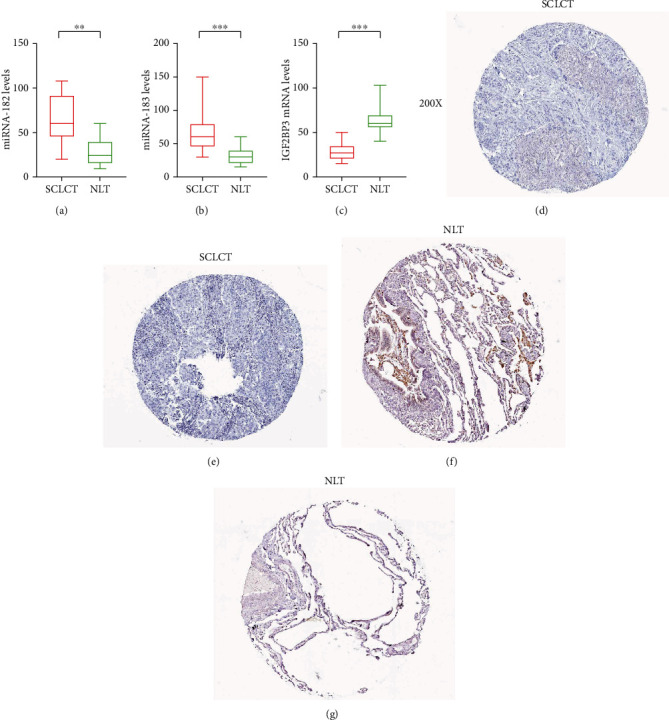
The expression level of hsa-miR-182, hsa-miR-183, and IGF2BP3 in normal lung tissues (NLT) and SCLC tissues (SCLCT). (a) Validation of has-miR-182 (^∗∗^*p* < 0.01). (b) Validation of hsa-miR-183 (^∗∗∗^*p* < 0.001). (c) Validation of IGF2BP3 (^∗∗∗^*p* < 0.001). (d, e) The expression level of IGF2BP3 in human SCLCT. (f, g) The expression level of IGF2BP3 in human NLT (scale bar: 200x).

To further assess the expression of hsa-miR-183, hsa-miR-182, and IGF2BP3, a total of 20 pairs of normal lung tissues (NLT) and SCLC tissues (SCLCT) were enrolled as a validation cohort. The qRT-PCR method was used to confirm the differential expression levels from participant's samples. Consistent with the microarray data, hsa-miR-183 and hsa-miR-182 were significantly upregulated (Figures 7(a) and 7(b)) and IGF2BP3 was downregulated (Figure 7(c)) between 20 pairs of NLTs and SCLCTs which indicated that hsa-miR-183, hsa-miR-182, and IGF2BP3 could be the candidate biomarkers for SCLC. HPA database (human protein atlas) is based on proteomics, transcriptomics, and system biology data, which can map tissues, cells, organs, and so on. It includes not only the protein expression of tumor tissues but also normal tissues and the survival curve of tumor patients. The database results show that IGF2BP3 was upregulated in SCLC tissues compared with normal tissues (Figures 7(d)–7(g)).

## 4. Discussion

According to the statistics of the World Health Organization (who), 3/5 people in the world die of cancer, diabetes, cardiovascular diseases, and chronic respiratory diseases, and cancer is one of the most important causes of death. China's cancer mortality is close to that of the United States, Britain, and France, but higher than that of Asian countries. In recent years, the global incidence of cancer has shown a high trend, with the number of new patients increasing from 17.2 million in 2016 to 19.3 million in 2020. It is estimated that the number of new cancer patients in the world will reach 20.2 million in 2022 [11, 12]. In this article, GSE19945 and GSE149507 were downloaded from the GEO database. KEGG research showed that DEGs were mainly enriched in 6 pathways including the MAPK signaling pathway, cGMP-PKG signaling pathway, autophagy, axon guidance, regulation of actin cytoskeleton, parathyroid hormone synthesis, secretion and action, ErbB signaling pathway, and neurotrophin signaling pathway, which were shown to affect migration and proliferation [13–16]. The MAPK signaling pathway and the cGMP-PKG signaling pathway are common signaling pathways closely associated with carcinoma, which will not be discussed here. As for the ErbB signaling pathway, identification of the role of ErbB (EGFR, HER2) in tumor radioresistance has led to attempts to sensitize tumors by inhibiting receptor signaling [17]. ErbB family members play a key role in responding to extracellular signals and initiating downstream signal cascades via effector pathways [18]. The activation of alternative ErbB signaling pathways (especially those involved in Heregulin and HER3) can lead to resistance to egfitinib in NSCLC patients [19]. The expression of E-cadherin molecules in many tumor cells including colorectal cancer and breast cancer is significantly reduced or absent. The decrease of E-cadherin expression level is significantly related to the malignancy of tumor cells. The expression level of E-cadherin in breast cancer cells with low malignancy was significantly higher than that in malignant tumor cells, and its expression level was directly proportional to the formation of glandular canaliculi [20–22]. In vitro experiments more clearly confirmed the relationship between E-cadherin molecule and tumor invasion ability. The tumor cells expressing E-cadherin in the culture state do not invade the matrix attached to the medium, but if the anti-E-cadherin antibody is added, the tumor cells will obtain the ability of infiltration. Tumor cells that do not express E-cadherin molecule show invasive ability in culture, but if the CD Na of E-cadherin molecule is transfected into tumor cells to express E-cadherin molecule, the tumor cells will lose their invasive ability [23, 24].

miRNA-mRNA network was conducted by using Cytoscape. Five miRNAs (microRNA-183, microRNA-9, microRNA-1, microRNA-30a, and microRNA-182) were selected for deep study. After that, 1915 target genes were achieved and 9 of them were differentially expressed in GSE149507 (LDB2, KIAA0101, IGF2BP3, HSPA2, HIST1H2AE, FHL1, CLIC5, CCNB1, and PRDX1). Some of these miRNAs and mRNAs have been proved to be related to tumorigenesis and development. For example, miRNA-183 is a member of the miRNA-96-182-183 cluster located in 7q, which is the preferred site for loss of heterozygosity, translocation, and amplification. It was reported to be overexpressed in many types of tumors, including hepatocellular cancer [25], prostate cancer [26], esophageal squamous cell carcinoma [27], and bladder carcinoma [28]. Moreover, overexpression of microRNA-183 can inhibit the apoptosis induced by serum deprivation. When the microRNA-183 inhibitor reduces the expression of miR-183, the inhibition of apoptosis can be rescued. In addition, microRNA-183 accelerated the cell process and promoted cell proliferation in the G1/S phase [27]. As for hsa-microRNA-9, it was low expressed in peripheral blood of patients with osteoporosis. miRNA-9-5p promotes the occurrence and development of osteoporosis by targeting Wnt3a, inhibiting osteogenesis, and promoting adipogenesis [29]. In non-small-cell lung cancer, it can inhibit the migration and enhance the effect of radiation of non-small-cell lung cancer cells, which indicated that microRNA-9 enhanced the radiosensitivity of non-small-cell lung cancer and was regulated by the methylation state of its promoter [30]. The microRNA-30 family consists of 5 highly conserved mature members (microRNA-30a, microRNA-30b, microRNA-30C, microRNA-30d, and microRNA-30e), which are located on different chromosomes or adjacent loci. miR-30a is located on chromosome 6q13 and comes from the intron transcription unit [31]. A previous study reported that the level of miR-30a-3p in tumor tissue of hepatocellular carcinoma patients decreased significantly, which was related to lymph node metastasis, distant metastasis, and poor prognosis. In addition, it also confirmed that miR-30a-3p may inhibit the malignant progression of hepatocellular carcinoma by regulating insulin-like growth factors [32]. Another study showed that miR-30a may play a tumor-suppressive role in colorectal carcinoma. miR-30a highlights the potential of miR-30a and metaherin in the treatment of colorectal carcinoma by reducing metaherin and inhibiting cell migration and invasion [33]. Based on the association between them, 9 microRNA-mRNA (microRNA-183, microRNA-9, microRNA-1, microRNA-30a, and microRNA-182) were selected.

IGF2BP3 is a carcinoembryonic protein, which is most expressed during embryogenesis, but it is almost not expressed in normal adult mouse tissues and adult tissues (except fibroblasts, lymphocytes and testis) [34]. Recent studies have shown that IGF2BP family members, including IGF2BP3, act as posttranscriptional regulatory factors of gene expression, which are associated with tumor cell proliferation, survival, chemotherapy resistance, and invasion [35]. At the same time, the clinical data also show that the expression of IGF2BP family members is upregulated in invasive malignant tumors and is related to the poor prognosis and metastasis of a variety of cancers [36–40]. Besides, previous study reported that IGF2BP3 can promote lung tumorigenesis via attenuating p53 protein stability. Therefore, we selected and tested whether microRNA-183, microRNA-182, and IGF2BP3 was differently expressed between NLTs and SCLCTs. qPCR results indicated that hsa-miR-182, hsa-miR-183, and IGF2BP3 were differentially expressed in NLTs and SCLCTs. Besides, IHC results indicated that IGF2BP3 had a significant difference in SCLCTs compared with NLTs. However, this manuscript lacks animal and cell experimental verification, so more functional and mechanism studies need to prove our conclusions.

The role of microRNA and mRNA in a variety of tumors is very important, because gene expression regulation may be a new choice for tumor therapy. Our study indicated that many differentially expressed genes were involved in the occurrence of SCLC. Therefore, suppression of hsa-miR-182, hsa-miR-183, and upregulated IGF2BP3 may have valuable worth in SCLC patients.

## 5. Conclusion

In this article, bioinformatics methods were used to analyze the differentially expressed genes in SCLC and normal tissues. qPCR and IHC results indicated that hsa-miR-182, hsa-miR-183, and IGF2BP3 were differentially expressed between NLTs and SCLCTs. However, we need more cell experiments to prove it.

## Figures and Tables

**Table 1:** Relationship between the expression level of IGF2BP3 and clinical features.

Characteristic	Low expression of IGF2BP3	High expression of IGF2BP3	*p*
*n*	518	519	
T stage, *n* (%)			<0.001
T1	178 (17.2%)	111 (10.7%)	
T2	260 (25.1%)	323 (31.2%)	
T3	56 (5.4%)	64 (6.2%)	
T4	21 (2%)	21 (2%)	
N stage, *n* (%)			<0.001
N0	357 (35.2%)	311 (30.6%)	
N1	87 (8.6%)	139 (13.7%)	
N2	56 (5.5%)	58 (5.7%)	
N3	1 (0.1%)	6 (0.6%)	
M stage, *n* (%)			0.184
M0	378 (47%)	395 (49.1%)	
M1	20 (2.5%)	12 (1.5%)	
Gender, *n* (%)			<0.001
Female	244 (23.5%)	173 (16.7%)	
Male	274 (26.4%)	346 (33.4%)	
Race, *n* (%)			0.526
Asian	7 (0.8%)	9 (1.1%)	
Black or African American	48 (5.6%)	37 (4.3%)	
White	386 (45%)	370 (43.2%)	
Age, median (IQR)	67 (60, 73)	67 (59, 73)	0.383

**Table 2:** Univariate analysis and multivariate analysis research the relationship between clinical characteristics (IGF2BP3) and prognosis of patients with SCLC.

Characteristics	Total(N)	Univariate analysis		Multivariate analysis	
		Hazard ratio (95% CI)	*p* value	Hazard ratio (95% CI)	*p* value
T stage	1019				
T1&T2	860	Reference			
T3&T4	159	1.889 (1.480-2.412)	<0.001	1.635 (1.243-2.152)	<0.001
N stage	1000				
N0&N1	882	Reference			
N2&N3	118	1.799 (1.372-2.357)	<0.001	1.859 (1.388-2.489)	<0.001
M stage	792				
M0	760	Reference			
M1	32	2.269 (1.439-3.577)	<0.001	1.946 (1.190-3.182)	0.008
Gender	1022				
Female	410	Reference			
Male	612	1.164 (0.949-1.428)	0.145		
Race	855				
Asian	16	Reference			
Black or African American &White	839	0.806 (0.333-1.952)	0.633		
IGF2BP3	1022	1.538 (1.951-2.234)	0.005	1.799 (2.242-3.387)	<0.001

## Data Availability

The datasets used and/or analyzed during the current study are available from the corresponding author on reasonable request.

## Abbreviations

GEO: Gene Expression Omnibus
KEGG: Kyoto Encyclopedia of Genes and Genomes
miRNA: MicroRNAs
TCGA: The Cancer Genome Atlas
qPCR: Quantitative real-time PCR
DEmiRNAs: Differentially expressed miRNAs
DEGs: Differentially expressed genes
SCLC: Small-cell lung cancer.
